# Supplementary material for: Smooth Interpolating Curves with Local Control and Monotone Alternating Curvature
Source: Comput Graph Forum. 2022 Oct 6;41(5):25–38. doi: 10.1111/cgf.14600 (PMC9827861; doi:10.1111/cgf.14600)
Supplement: Supplementary file 1 — Supplement Material [file CGF-41-25-s001.zip › Local-Smooth-Interpolating-MonoCurvature/extern/clothoids/docs/api-cpp/program_listing_file_Fresnel.cc.html]

Program Listing for File Fresnel.cc — Clothoids v2.0.9

### Navigation

- index
- toc
- Clothoids »
- Program Listing for File Fresnel.cc

# Program Listing for File Fresnel.cc¶

↰ Return to documentation for file (`Fresnel.cc`)

```
/*--------------------------------------------------------------------------*\
 |                                                                          |
 |  Copyright (C) 2017                                                      |
 |                                                                          |
 |         , __                 , __                                        |
 |        /|/  \               /|/  \                                       |
 |         | __/ _   ,_         | __/ _   ,_                                |
 |         |   \|/  /  |  |   | |   \|/  /  |  |   |                        |
 |         |(__/|__/   |_/ \_/|/|(__/|__/   |_/ \_/|/                       |
 |                           /|                   /|                        |
 |                           \|                   \|                        |
 |                                                                          |
 |      Enrico Bertolazzi                                                   |
 |      Dipartimento di Ingegneria Industriale                              |
 |      Universita` degli Studi di Trento                                   |
 |      email: enrico.bertolazzi@unitn.it                                   |
 |                                                                          |
\*--------------------------------------------------------------------------*/

#include "Clothoids.hh"
#include "PolynomialRoots.hh"

#ifndef DOXYGEN_SHOULD_SKIP_THIS
#define A_THRESOLD   0.01
#define A_SERIE_SIZE 3
#endif

#ifdef __GNUC__
#pragma GCC diagnostic push
#pragma GCC diagnostic ignored "-Wshadow"
#endif
#ifdef __clang__
#pragma clang diagnostic push
#pragma clang diagnostic ignored "-Wshadow"
#pragma clang diagnostic ignored "-Wglobal-constructors"
#endif

// Workaround for Visual Studio
#ifdef min
  #undef min
#endif

#ifdef max
  #undef max
#endif

#include <cmath>
#include <cfloat>
#include <algorithm>

namespace G2lib {

  using std::abs;
  using std::min;
  using std::max;

  #ifndef DOXYGEN_SHOULD_SKIP_THIS

  /*
  // This function calculates the fresnel cosine and sine integrals.
  // Input:
  // y = values for which fresnel integrals have to be evaluated
  //
  // Output:
  // FresnelC = fresnel cosine integral of y
  // FresnelS = fresnel sine integral of y
  //
  // Adapted from:
  // Atlas for computing mathematical functions : an illustrated guide for
  // practitioners, with programs in C and Mathematica / William J. Thompson.
  // New York : Wiley, c1997.
  //
  // Author: Venkata Sivakanth Telasula
  // email: sivakanth.telasula@gmail.com
  // date: August 11, 2005
  */
  static const real_type fn[] = {
    0.49999988085884732562,
    1.3511177791210715095,
    1.3175407836168659241,
    1.1861149300293854992,
    0.7709627298888346769,
    0.4173874338787963957,
    0.19044202705272903923,
    0.06655998896627697537,
    0.022789258616785717418,
    0.0040116689358507943804,
    0.0012192036851249883877
  };

  static const real_type fd[] = {
    1.0,
    2.7022305772400260215,
    4.2059268151438492767,
    4.5221882840107715516,
    3.7240352281630359588,
    2.4589286254678152943,
    1.3125491629443702962,
    0.5997685720120932908,
    0.20907680750378849485,
    0.07159621634657901433,
    0.012602969513793714191,
    0.0038302423512931250065
  };

  static const real_type gn[] = {
    0.50000014392706344801,
    0.032346434925349128728,
    0.17619325157863254363,
    0.038606273170706486252,
    0.023693692309257725361,
    0.007092018516845033662,
    0.0012492123212412087428,
    0.00044023040894778468486,
    -8.80266827476172521e-6,
    -1.4033554916580018648e-8,
    2.3509221782155474353e-10
  };

  static const real_type gd[] = {
    1.0,
    2.0646987497019598937,
    2.9109311766948031235,
    2.6561936751333032911,
    2.0195563983177268073,
    1.1167891129189363902,
    0.57267874755973172715,
    0.19408481169593070798,
    0.07634808341431248904,
    0.011573247407207865977,
    0.0044099273693067311209,
    -0.00009070958410429993314
  };

  #endif

  /*
  //  #######
  //  #       #####  ######  ####  #    # ###### #
  //  #       #    # #      #      ##   # #      #
  //  #####   #    # #####   ####  # #  # #####  #
  //  #       #####  #           # #  # # #      #
  //  #       #   #  #      #    # #   ## #      #
  //  #       #    # ######  ####  #    # ###### ######
  */

  void
  FresnelCS( real_type y, real_type & C, real_type & S ) {

    real_type const eps = 1E-15;
    real_type const x   = y > 0 ? y : -y;

    if ( x < 1.0 ) {
      real_type twofn, fact, denterm, numterm, sum, term;

      real_type const s = Utils::m_pi_2*(x*x);
      real_type const t = -s*s;

      // Cosine integral series
      twofn   =  0.0;
      fact    =  1.0;
      denterm =  1.0;
      numterm =  1.0;
      sum     =  1.0;
      do {
        twofn   += 2.0;
        fact    *= twofn*(twofn-1.0);
        denterm += 4.0;
        numterm *= t;
        term     = numterm/(fact*denterm);
        sum     += term;
      } while ( abs(term) > eps*abs(sum) );

      C = x*sum;

      // Sine integral series
      twofn   = 1.0;
      fact    = 1.0;
      denterm = 3.0;
      numterm = 1.0;
      sum     = 1.0/3.0;
      do {
        twofn   += 2.0;
        fact    *= twofn*(twofn-1.0);
        denterm += 4.0;
        numterm *= t;
        term     = numterm/(fact*denterm);
        sum     += term;
      } while ( abs(term) > eps*abs(sum) );

      S = Utils::m_pi_2*sum*(x*x*x);

    } else if ( x < 6.0 ) {

      // Rational approximation for f
      real_type sumn = 0.0;
      real_type sumd = fd[11];
      for ( int_type k=10; k >= 0; --k ) {
        sumn = fn[k] + x*sumn;
        sumd = fd[k] + x*sumd;
      }
      real_type f = sumn/sumd;

      // Rational approximation for g
      sumn = 0.0;
      sumd = gd[11];
      for ( int_type k=10; k >= 0; --k ) {
        sumn = gn[k] + x*sumn;
        sumd = gd[k] + x*sumd;
      }
      real_type g = sumn/sumd;

      real_type U    = Utils::m_pi_2*(x*x);
      real_type SinU = sin(U);
      real_type CosU = cos(U);
      C = 0.5 + f*SinU - g*CosU;
      S = 0.5 - f*CosU - g*SinU;

    } else {

      real_type absterm;

      // x >= 6; asymptotic expansions for  f  and  g

      real_type const s = Utils::m_pi*x*x;
      real_type const t = -1/(s*s);

      // Expansion for f
      real_type numterm = -1.0;
      real_type term    =  1.0;
      real_type sum     =  1.0;
      real_type oldterm =  1.0;
      real_type eps10   =  0.1 * eps;

      do {
        numterm += 4.0;
        term    *= numterm*(numterm-2.0)*t;
        sum     += term;
        absterm  = abs(term);
        UTILS_ASSERT(
          oldterm >= absterm,
          "In FresnelCS f not converged to eps, x = {} oldterm = {} absterm = {}\n",
          x, oldterm, absterm
        );
        oldterm  = absterm;
      } while ( absterm > eps10 * abs(sum) );

      real_type f = sum / (Utils::m_pi*x);

      //  Expansion for  g
      numterm = -1.0;
      term    =  1.0;
      sum     =  1.0;
      oldterm =  1.0;

      do {
        numterm += 4.0;
        term    *= numterm*(numterm+2.0)*t;
        sum     += term;
        absterm  = abs(term);
        UTILS_ASSERT(
          oldterm >= absterm,
          "In FresnelCS g not converged to eps, x = {} oldterm = {} absterm = {}\n",
          x, oldterm, absterm
        );
        oldterm  = absterm;
      } while ( absterm > eps10 * abs(sum) );

      real_type g = Utils::m_pi*x; g = sum/(g*g*x);

      real_type U    = Utils::m_pi_2*(x*x);
      real_type SinU = sin(U);
      real_type CosU = cos(U);
      C = 0.5 + f*SinU - g*CosU;
      S = 0.5 - f*CosU - g*SinU;

    }
    if ( y < 0 ) { C = -C; S = -S; }
  }

  // -------------------------------------------------------------------------
  // -------------------------------------------------------------------------

  void
  FresnelCS(
    int_type    nk,
    real_type   t,
    real_type * C,
    real_type * S
  ) {
    FresnelCS(t,C[0],S[0]);
    if ( nk > 1 ) {
      real_type tt = Utils::m_pi_2*(t*t);
      real_type ss = sin(tt);
      real_type cc = cos(tt);
      C[1] = ss*Utils::m_1_pi;
      S[1] = (1-cc)*Utils::m_1_pi;
      if ( nk > 2 ) {
        C[2] = (t*ss-S[0])*Utils::m_1_pi;
        S[2] = (C[0]-t*cc)*Utils::m_1_pi;
      }
    }
  }

  // -------------------------------------------------------------------------
  // -------------------------------------------------------------------------

  #ifndef DOXYGEN_SHOULD_SKIP_THIS
  static
  void
  evalXYaLarge(
    real_type   a,
    real_type   b,
    real_type & X,
    real_type & Y
  ) {
    real_type s    = a > 0 ? +1 : -1;
    real_type absa = abs(a);
    real_type z    = m_1_sqrt_pi*sqrt(absa);
    real_type ell  = s*b*m_1_sqrt_pi/sqrt(absa);
    real_type g    = -0.5*s*(b*b)/absa;
    real_type cg   = cos(g)/z;
    real_type sg   = sin(g)/z;

    real_type Cl, Sl, Cz, Sz;
    FresnelCS( ell,   Cl, Sl );
    FresnelCS( ell+z, Cz, Sz );

    real_type dC0 = Cz - Cl;
    real_type dS0 = Sz - Sl;

    X = cg * dC0 - s * sg * dS0;
    Y = sg * dC0 + s * cg * dS0;
  }

  // -------------------------------------------------------------------------
  // nk max 3
  static
  void
  evalXYaLarge(
    int_type    nk,
    real_type   a,
    real_type   b,
    real_type * X,
    real_type * Y
  ) {

    UTILS_ASSERT(
      nk < 4 && nk > 0,
      "In evalXYaLarge first argument nk must be in 1..3, nk {}\n", nk
    );

    real_type s    = a > 0 ? +1 : -1;
    real_type absa = abs(a);
    real_type z    = m_1_sqrt_pi*sqrt(absa);
    real_type ell  = s*b*m_1_sqrt_pi/sqrt(absa);
    real_type g    = -0.5*s*(b*b)/absa;
    real_type cg   = cos(g)/z;
    real_type sg   = sin(g)/z;

    real_type Cl[3], Sl[3], Cz[3], Sz[3];

    FresnelCS( nk, ell,   Cl, Sl );
    FresnelCS( nk, ell+z, Cz, Sz );

    real_type dC0 = Cz[0] - Cl[0];
    real_type dS0 = Sz[0] - Sl[0];
    X[0] = cg * dC0 - s * sg * dS0;
    Y[0] = sg * dC0 + s * cg * dS0;
    if ( nk > 1 ) {
      cg /= z;
      sg /= z;
      real_type dC1 = Cz[1] - Cl[1];
      real_type dS1 = Sz[1] - Sl[1];
      real_type DC  = dC1-ell*dC0;
      real_type DS  = dS1-ell*dS0;
      X[1] = cg * DC - s * sg * DS;
      Y[1] = sg * DC + s * cg * DS;
      if ( nk > 2 ) {
        real_type dC2 = Cz[2] - Cl[2];
        real_type dS2 = Sz[2] - Sl[2];
        DC   = dC2+ell*(ell*dC0-2*dC1);
        DS   = dS2+ell*(ell*dS0-2*dS1);
        cg   = cg/z;
        sg   = sg/z;
        X[2] = cg * DC - s * sg * DS;
        Y[2] = sg * DC + s * cg * DS;
      }
    }
  }

  // -------------------------------------------------------------------------
  // -------------------------------------------------------------------------

  static
  real_type
  LommelReduced( real_type mu, real_type nu, real_type b ) {
    real_type tmp = 1/((mu+nu+1)*(mu-nu+1));
    real_type res = tmp;
    for ( int_type n = 1; n <= 100; ++n ) {
      tmp *= (-b/(2*n+mu-nu+1)) * (b/(2*n+mu+nu+1));
      res += tmp;
      if ( abs(tmp) < abs(res) * 1e-50 ) break;
    }
    return res;
  }

  // -------------------------------------------------------------------------
  // -------------------------------------------------------------------------

  static
  void
  evalXYazero(
    int_type    nk,
    real_type   b,
    real_type * X,
    real_type * Y
  ) {
    real_type sb = sin(b);
    real_type cb = cos(b);
    real_type b2 = b*b;
    if ( abs(b) < 1e-3 ) {
      X[0] = 1-(b2/6)*(1-(b2/20)*(1-(b2/42)));
      Y[0] = (b/2)*(1-(b2/12)*(1-(b2/30)));
    } else {
      X[0] = sb/b;
      Y[0] = (1-cb)/b;
    }
    // use recurrence in the stable part
    int_type m = int_type(floor(2*b));
    if ( m >= nk ) m = nk-1;
    if ( m < 1   ) m = 1;
    for ( int_type k = 1; k < m; ++k ) {
      X[k] = (sb-k*Y[k-1])/b;
      Y[k] = (k*X[k-1]-cb)/b;
    }
    //  use Lommel for the unstable part
    if ( m < nk ) {
      real_type A   = b*sb;
      real_type D   = sb-b*cb;
      real_type B   = b*D;
      real_type C   = -b2*sb;
      real_type rLa = LommelReduced(m+0.5,1.5,b);
      real_type rLd = LommelReduced(m+0.5,0.5,b);
      for ( int_type k = m; k < nk; ++k ) {
        real_type rLb = LommelReduced(k+1.5,0.5,b);
        real_type rLc = LommelReduced(k+1.5,1.5,b);
        X[k] = ( k*A*rLa + B*rLb + cb ) / (1+k);
        Y[k] = ( C*rLc + sb ) / (2+k) + D*rLd;
          rLa  = rLc;
        rLd  = rLb;
      }
    }
  }

  // -------------------------------------------------------------------------
  // -------------------------------------------------------------------------

  static
  void
  evalXYaSmall(
    real_type   a,
    real_type   b,
    int_type    p,
    real_type & X,
    real_type & Y
  ) {

    UTILS_ASSERT(
      p < 11 && p > 0, "In evalXYaSmall p = {} must be in 1..10\n", p
    );

    real_type X0[43], Y0[43];

    int_type nkk = 4*p + 3; // max 43
    evalXYazero( nkk, b, X0, Y0 );

    X = X0[0]-(a/2)*Y0[2];
    Y = Y0[0]+(a/2)*X0[2];

    real_type t  = 1;
    real_type aa = -a*a/4; // controllare!
    for ( int_type n=1; n <= p; ++n ) {
      t *= aa/(2*n*(2*n-1));
      real_type bf = a/(4*n+2);
      int_type  jj = 4*n;
      X += t*(X0[jj]-bf*Y0[jj+2]);
      Y += t*(Y0[jj]+bf*X0[jj+2]);
    }
  }

  // -------------------------------------------------------------------------

  static
  void
  evalXYaSmall(
    int_type    nk,
    real_type   a,
    real_type   b,
    int_type    p,
    real_type * X,
    real_type * Y
  ) {

    int_type  nkk = nk + 4*p + 2; // max 45
    real_type X0[45], Y0[45];

    UTILS_ASSERT(
      nkk < 46,
      "In evalXYaSmall (nk,p) = ({},{})\n"
      "nk + 4*p + 2 = {} must be less than 46\n",
      nk, p, nkk
    );

    evalXYazero( nkk, b, X0, Y0 );

    for ( int_type j=0; j < nk; ++j ) {
      X[j] = X0[j]-(a/2)*Y0[j+2];
      Y[j] = Y0[j]+(a/2)*X0[j+2];
    }

    real_type t  = 1;
    real_type aa = -a*a/4; // controllare!
    for ( int_type n=1; n <= p; ++n ) {
      t *= aa/(2*n*(2*n-1));
      real_type bf = a/(4*n+2);
      for ( int_type j = 0; j < nk; ++j ) {
        int_type jj = 4*n+j;
        X[j] += t*(X0[jj]-bf*Y0[jj+2]);
        Y[j] += t*(Y0[jj]+bf*X0[jj+2]);
      }
    }
  }
  #endif

  // -------------------------------------------------------------------------
  // -------------------------------------------------------------------------

  void
  GeneralizedFresnelCS(
    real_type   a,
    real_type   b,
    real_type   c,
    real_type & intC,
    real_type & intS
  ) {
    real_type xx, yy;
    if ( abs(a) < A_THRESOLD ) evalXYaSmall( a, b, A_SERIE_SIZE, xx, yy );
    else                       evalXYaLarge( a, b, xx, yy );

    real_type cosc = cos(c);
    real_type sinc = sin(c);

    intC = xx * cosc - yy * sinc;
    intS = xx * sinc + yy * cosc;
  }

  // -------------------------------------------------------------------------
  // -------------------------------------------------------------------------

  void
  GeneralizedFresnelCS(
    int_type    nk,
    real_type   a,
    real_type   b,
    real_type   c,
    real_type * intC,
    real_type * intS
  ) {
    UTILS_ASSERT( nk > 0 && nk < 4, "nk = {} must be in 1..3\n", nk );

    if ( abs(a) < A_THRESOLD ) evalXYaSmall( nk, a, b, A_SERIE_SIZE, intC, intS );
    else                       evalXYaLarge( nk, a, b, intC, intS );

    real_type cosc = cos(c);
    real_type sinc = sin(c);

    for ( int_type k = 0; k < nk; ++k ) {
      real_type xx = intC[k];
      real_type yy = intS[k];
      intC[k] = xx * cosc - yy * sinc;
      intS[k] = xx * sinc + yy * cosc;
    }
  }

  #ifndef DOXYGEN_SHOULD_SKIP_THIS

  // -------------------------------------------------------------------------

  void
  ClothoidData::nor_ISO(
    real_type   s,
    real_type & nx,
    real_type & ny
  ) const {
    this->tg( s, ny, nx ); nx = -nx;
  }

  void
  ClothoidData::nor_SAE(
    real_type   s,
    real_type & nx,
    real_type & ny
  ) const {
    this->tg( s, ny, nx ); ny = -ny;
  }

  // - - - - - - - - - - - - - - - - - - - - - - - - - - - - - - - - - - - - - -

  void
  ClothoidData::nor_ISO_D(
    real_type   s,
    real_type & nx_D,
    real_type & ny_D
  ) const {
    this->tg_D( s, ny_D, nx_D ); nx_D = -nx_D;
  }

  void
  ClothoidData::nor_SAE_D(
    real_type   s,
    real_type & nx_D,
    real_type & ny_D
  ) const {
    this->tg_D( s, ny_D, nx_D ); ny_D = -ny_D;
  }

  // - - - - - - - - - - - - - - - - - - - - - - - - - - - - - - - - - - - - - -

  void
  ClothoidData::nor_ISO_DD(
    real_type   s,
    real_type & nx_DD,
    real_type & ny_DD
  ) const {
    this->tg_DD( s, ny_DD, nx_DD ); nx_DD = -nx_DD;
  }

  void
  ClothoidData::nor_SAE_DD(
    real_type   s,
    real_type & nx_DD,
    real_type & ny_DD
  ) const {
    this->tg_DD( s, ny_DD, nx_DD ); ny_DD = -ny_DD;
  }

  // - - - - - - - - - - - - - - - - - - - - - - - - - - - - - - - - - - - - - -

  void
  ClothoidData::nor_ISO_DDD(
    real_type   s,
    real_type & nx_DDD,
    real_type & ny_DDD
  ) const {
    this->tg_DDD( s, ny_DDD, nx_DDD ); nx_DDD = -nx_DDD;
  }

  void
  ClothoidData::nor_SAE_DDD(
    real_type   s,
    real_type & nx_DDD,
    real_type & ny_DDD
  ) const {
    this->tg_DDD( s, ny_DDD, nx_DDD ); ny_DDD = -ny_DDD;
  }

  // - - - - - - - - - - - - - - - - - - - - - - - - - - - - - - - - - - - - - -

  real_type
  ClothoidData::tg_x_D( real_type s ) const {
    return -sin(theta(s)) * theta_D(s);
  }

  // - - - - - - - - - - - - - - - - - - - - - - - - - - - - - - - - - - - - - -

  real_type
  ClothoidData::tg_y_D( real_type s ) const {
    return cos(theta(s)) * theta_D(s);
  }

  // - - - - - - - - - - - - - - - - - - - - - - - - - - - - - - - - - - - - - -

  real_type
  ClothoidData::tg_x_DD( real_type s ) const {
    real_type th    = theta(s);
    real_type th_D  = theta_D(s);
    real_type th_DD = theta_DD(s);
    real_type S     = sin(th);
    real_type C     = cos(th);
    return -C*th_D*th_D-S*th_DD;
  }

  // - - - - - - - - - - - - - - - - - - - - - - - - - - - - - - - - - - - - - -

  real_type
  ClothoidData::tg_y_DD( real_type s ) const {
    real_type th    = theta(s);
    real_type th_D  = theta_D(s);
    real_type th_DD = theta_DD(s);
    real_type S     = sin(th);
    real_type C     = cos(th);
    return -S*th_D*th_D+C*th_DD;
  }

  // - - - - - - - - - - - - - - - - - - - - - - - - - - - - - - - - - - - - - -

  real_type
  ClothoidData::tg_x_DDD( real_type s ) const {
    real_type th    = theta(s);
    real_type th_D  = theta_D(s);
    real_type th_DD = theta_DD(s);
    real_type S     = sin(th);
    real_type C     = cos(th);
    real_type th_D2 = th_D*th_D;
    return th_D*(S*th_D2-C*th_DD*(2*th_D-1));
  }

  // - - - - - - - - - - - - - - - - - - - - - - - - - - - - - - - - - - - - - -

  real_type
  ClothoidData::tg_y_DDD( real_type s ) const {
    real_type th    = theta(s);
    real_type th_D  = theta_D(s);
    real_type th_DD = theta_DD(s);
    real_type S     = sin(th);
    real_type C     = cos(th);
    real_type th_D2 = th_D*th_D;
    return -th_D*(C*th_D2+S*th_DD*(2*th_D+1));
  }

  // - - - - - - - - - - - - - - - - - - - - - - - - - - - - - - - - - - - - - -

  void
  ClothoidData::tg(
    real_type   s,
    real_type & tx,
    real_type & ty
  ) const {
    real_type th = theta(s);
    tx = cos(th);
    ty = sin(th);
  }

  // - - - - - - - - - - - - - - - - - - - - - - - - - - - - - - - - - - - - - -

  void
  ClothoidData::tg_D(
    real_type   s,
    real_type & tx_D,
    real_type & ty_D
  ) const {
    real_type th   = theta(s);
    real_type th_D = theta_D(s);
    tx_D =  sin(th)*th_D;
    ty_D = -cos(th)*th_D;
  }

  // - - - - - - - - - - - - - - - - - - - - - - - - - - - - - - - - - - - - - -

  void
  ClothoidData::tg_DD(
    real_type   s,
    real_type & tx_DD,
    real_type & ty_DD
  ) const {
    real_type th    = theta(s);
    real_type th_D  = theta_D(s);
    real_type th_DD = theta_DD(s);
    real_type S     = sin(th);
    real_type C     = cos(th);
    tx_DD = C*th_D*th_D+S*th_DD;
    ty_DD = S*th_D*th_D-C*th_DD;
  }

  // - - - - - - - - - - - - - - - - - - - - - - - - - - - - - - - - - - - - - -

  void
  ClothoidData::tg_DDD(
    real_type   s,
    real_type & tx_DDD,
    real_type & ty_DDD
  ) const {
    real_type th    = theta(s);
    real_type th_D  = theta_D(s);
    real_type th_DD = theta_DD(s);
    real_type S     = sin(th);
    real_type C     = cos(th);
    real_type th_D2 = th_D*th_D;
    tx_DDD = th_D*(C*th_DD*(2*th_D-1)-S*th_D2);
    ty_DDD = th_D*(C*th_D2+S*th_DD*(2*th_D+1));
  }

  // - - - - - - - - - - - - - - - - - - - - - - - - - - - - - - - - - - - - - -

  real_type
  ClothoidData::X( real_type s ) const {
    real_type C, S;
    GeneralizedFresnelCS( dk*s*s, kappa0*s, theta0, C, S );
    return x0 + s*C;
  }

  // - - - - - - - - - - - - - - - - - - - - - - - - - - - - - - - - - - - - - -

  real_type
  ClothoidData::Y( real_type s ) const {
    real_type C, S;
    GeneralizedFresnelCS( dk*s*s, kappa0*s, theta0, C, S );
    return y0 + s*S;
  }

  // - - - - - - - - - - - - - - - - - - - - - - - - - - - - - - - - - - - - - -

  real_type
  ClothoidData::X_D( real_type s ) const
  { return cos( theta(s) ); }

  // - - - - - - - - - - - - - - - - - - - - - - - - - - - - - - - - - - - - - -

  real_type
  ClothoidData::Y_D( real_type s ) const
  { return sin( theta(s) ); }

  // - - - - - - - - - - - - - - - - - - - - - - - - - - - - - - - - - - - - - -

  real_type
  ClothoidData::X_DD( real_type s ) const {
    real_type theta   = theta0 + s*(kappa0+0.5*s*dk);
    real_type theta_D = kappa0 + s*dk;
    return -sin(theta)*theta_D;
  }

  // - - - - - - - - - - - - - - - - - - - - - - - - - - - - - - - - - - - - - -

  real_type
  ClothoidData::Y_DD( real_type s ) const {
    real_type theta   = theta0 + s*(kappa0+0.5*s*dk);
    real_type theta_D = kappa0 + s*dk;
    return cos(theta)*theta_D;
  }

  // - - - - - - - - - - - - - - - - - - - - - - - - - - - - - - - - - - - - - -

  real_type
  ClothoidData::X_DDD( real_type s ) const {
    real_type theta   = theta0 + s*(kappa0+0.5*s*dk);
    real_type theta_D = kappa0+s*dk;
    return -cos(theta)*theta_D*theta_D-sin(theta)*dk;
  }

  // - - - - - - - - - - - - - - - - - - - - - - - - - - - - - - - - - - - - - -

  real_type
  ClothoidData::Y_DDD( real_type s ) const {
    real_type theta   = theta0 + s*(kappa0+0.5*s*dk);
    real_type theta_D = kappa0+s*dk;
    return -sin(theta)*theta_D*theta_D+cos(theta)*dk;
  }

  // - - - - - - - - - - - - - - - - - - - - - - - - - - - - - - - - - - - - - -

  real_type
  ClothoidData::X_ISO( real_type s, real_type offs ) const
  { return X(s) + offs * nor_x_ISO(s); }

  real_type
  ClothoidData::Y_ISO( real_type s, real_type offs ) const
  { return Y(s) + offs * nor_y_ISO(s); }

  real_type
  ClothoidData::X_ISO_D( real_type s, real_type offs ) const
  { return X_D(s) + offs * nor_x_ISO_D(s); }

  real_type
  ClothoidData::Y_ISO_D( real_type s, real_type offs ) const
  { return Y_D(s) + offs * nor_y_ISO_D(s); }

  real_type
  ClothoidData::X_ISO_DD( real_type s, real_type offs ) const
  { return X_DD(s) + offs * nor_x_ISO_DD(s); }

  real_type
  ClothoidData::Y_ISO_DD( real_type s, real_type offs ) const
  { return Y_DD(s) + offs * nor_y_ISO_DD(s); }

  real_type
  ClothoidData::X_ISO_DDD( real_type s, real_type offs ) const
  { return X_DDD(s) + offs * nor_x_ISO_DDD(s); }

  real_type
  ClothoidData::Y_ISO_DDD( real_type s, real_type offs ) const
  { return Y_DDD(s) + offs * nor_y_ISO_DDD(s); }

  // - - - - - - - - - - - - - - - - - - - - - - - - - - - - - - - -

  real_type
  ClothoidData::X_SAE( real_type s, real_type offs ) const
  { return X(s) + offs * nor_x_SAE(s); }

  real_type
  ClothoidData::Y_SAE( real_type s, real_type offs ) const
  { return Y(s) + offs * nor_y_SAE(s); }

  real_type
  ClothoidData::X_SAE_D( real_type s, real_type offs ) const
  { return X_D(s) + offs * nor_x_SAE_D(s); }

  real_type
  ClothoidData::Y_SAE_D( real_type s, real_type offs ) const
  { return Y_D(s) + offs * nor_y_SAE_D(s); }

  real_type
  ClothoidData::X_SAE_DD( real_type s, real_type offs ) const
  { return X_DD(s) + offs * nor_x_SAE_DD(s); }

  real_type
  ClothoidData::Y_SAE_DD( real_type s, real_type offs ) const
  { return Y_DD(s) + offs * nor_y_SAE_DD(s); }

  real_type
  ClothoidData::X_SAE_DDD( real_type s, real_type offs ) const
  { return X_DDD(s) + offs * nor_x_SAE_DDD(s); }

  real_type
  ClothoidData::Y_SAE_DDD( real_type s, real_type offs ) const
  { return Y_DDD(s) + offs * nor_y_SAE_DDD(s); }

  // - - - - - - - - - - - - - - - - - - - - - - - - - - - - - - - -

  void
  ClothoidData::evaluate(
    real_type   s,
    real_type & theta,
    real_type & kappa,
    real_type & x,
    real_type & y
  ) const {
    real_type C, S;
    GeneralizedFresnelCS( dk*s*s, kappa0*s, theta0, C, S );
    x     = x0 + s*C;
    y     = y0 + s*S;
    theta = theta0 + s*(kappa0+0.5*s*dk);
    kappa = kappa0 + s*dk;
  }

  // - - - - - - - - - - - - - - - - - - - - - - - - - - - - - - - - - - - - - -

  void
  ClothoidData::eval(
    real_type   s,
    real_type & x,
    real_type & y
  ) const {
    real_type C, S;
    GeneralizedFresnelCS( dk*s*s, kappa0*s, theta0, C, S );
    x = x0 + s*C;
    y = y0 + s*S;
  }

  // - - - - - - - - - - - - - - - - - - - - - - - - - - - - - - - - - - - - - -

  void
  ClothoidData::eval_D(
    real_type   s,
    real_type & x_D,
    real_type & y_D
  ) const {
    real_type theta = theta0 + s*(kappa0+0.5*s*dk);
    x_D = cos(theta);
    y_D = sin(theta);
  }

  // - - - - - - - - - - - - - - - - - - - - - - - - - - - - - - - - - - - - - -

  void
  ClothoidData::eval_DD(
    real_type   s,
    real_type & x_DD,
    real_type & y_DD
  ) const {
    real_type theta   = theta0 + s*(kappa0+0.5*s*dk);
    real_type theta_D = kappa0 + s*dk;
    x_DD = -sin(theta)*theta_D;
    y_DD =  cos(theta)*theta_D;
  }

  // - - - - - - - - - - - - - - - - - - - - - - - - - - - - - - - - - - - - - -

  void
  ClothoidData::eval_DDD(
    real_type   s,
    real_type & x_DDD,
    real_type & y_DDD
  ) const {
    real_type theta   = theta0 + s*(kappa0+0.5*s*dk);
    real_type theta_D = kappa0+s*dk;
    real_type C       = cos(theta);
    real_type S       = sin(theta);
    real_type th2     = theta_D*theta_D;
    x_DDD = -C*th2-S*dk;
    y_DDD = -S*th2+C*dk;
  }

  // - - - - - - - - - - - - - - - - - - - - - - - - - - - - - - - - - - - - - -

  void
  ClothoidData::eval_ISO(
    real_type   s,
    real_type   offs,
    real_type & x,
    real_type & y
  ) const {
    real_type C, S;
    GeneralizedFresnelCS( dk*s*s, kappa0*s, theta0, C, S );
    real_type theta = theta0 + s*(kappa0+0.5*s*dk);
    real_type tx    = cos( theta );
    real_type ty    = sin( theta );
    real_type nx    = -ty;
    real_type ny    = tx;
    x = x0 + s*C + offs * nx;
    y = y0 + s*S + offs * ny;
  }

  // - - - - - - - - - - - - - - - - - - - - - - - - - - - - - - - - - - - - - -

  void
  ClothoidData::eval_ISO_D(
    real_type   s,
    real_type   offs,
    real_type & x_D,
    real_type & y_D
  ) const {
    real_type theta   = theta0 + s*(kappa0+0.5*s*dk);
    real_type theta_D = kappa0 + s*dk;
    real_type scale   = 1-offs*theta_D;
    x_D = cos(theta)*scale;
    y_D = sin(theta)*scale;
  }

  // - - - - - - - - - - - - - - - - - - - - - - - - - - - - - - - - - - - - - -

  void
  ClothoidData::eval_ISO_DD(
    real_type   s,
    real_type   offs,
    real_type & x_DD,
    real_type & y_DD
  ) const {
    real_type theta   = theta0 + s*(kappa0+0.5*s*dk);
    real_type theta_D = kappa0 + s*dk;
    real_type C       = cos(theta);
    real_type S       = sin(theta);
    real_type tmp1    = theta_D*(1-theta_D*offs);
    real_type tmp2    = -offs*dk;
    x_DD = -tmp1*S + C*tmp2;
    y_DD =  tmp1*C + S*tmp2;
  }

  // - - - - - - - - - - - - - - - - - - - - - - - - - - - - - - - - - - - - - -

  void
  ClothoidData::eval_ISO_DDD(
    real_type   s,
    real_type   offs,
    real_type & x_DDD,
    real_type & y_DDD
  ) const {
    real_type theta   = theta0 + s*(kappa0+0.5*s*dk);
    real_type theta_D = kappa0 + s*dk;
    real_type C       = cos(theta);
    real_type S       = sin(theta);
    real_type tmp0    = -theta_D*offs;
    real_type tmp1    = -theta_D*theta_D*(1+tmp0);
    real_type tmp2    = dk*(1+3*tmp0);
    x_DDD = tmp1*C-tmp2*S;
    y_DDD = tmp1*S+tmp2*C;
  }

  // - - - - - - - - - - - - - - - - - - - - - - - - - - - - - - - - - - - - - -

  void
  ClothoidData::Pinfinity( real_type & x, real_type & y, bool plus ) const {
    real_type theta, tmp;
    this->evaluate( -kappa0/dk, theta, tmp, x, y );
    real_type Ct = cos(theta);
    real_type St = sin(theta);
    tmp = 0.5*sqrt( Utils::m_pi/abs(dk) );
    if ( !plus ) tmp = -tmp;
    if ( dk > 0 ) {
      x += tmp*(Ct-St);
      y += tmp*(St+Ct);
    } else {
      x += tmp*(Ct+St);
      y += tmp*(St-Ct);
    }
  }

  // - - - - - - - - - - - - - - - - - - - - - - - - - - - - - - - - - - - - - -

  void
  ClothoidData::eval( real_type s, ClothoidData & C ) const {
    this->evaluate( s, C.theta0, C.kappa0, C.x0, C.y0 );
    C.dk = dk;
  }

  // - - - - - - - - - - - - - - - - - - - - - - - - - - - - - - - - - - - - - -

  void
  ClothoidData::reverse( real_type L ) {
    real_type C, S;
    GeneralizedFresnelCS( dk*L*L, kappa0*L, theta0, C, S );
    x0     += L*C;
    y0     += L*S;
    theta0 += L*(kappa0+0.5*L*dk);
    kappa0 += L*dk;
    theta0 += Utils::m_pi;
    while ( theta0 >  Utils::m_pi ) theta0 -= Utils::m_2pi;
    while ( theta0 < -Utils::m_pi ) theta0 += Utils::m_2pi;
    kappa0  = -kappa0;
  }

  // - - - - - - - - - - - - - - - - - - - - - - - - - - - - - - - - - - - - - -

  void
  ClothoidData::reverse( real_type L, ClothoidData & out ) const {
    this->evaluate( L, out.theta0, out.kappa0, out.x0, out.y0 );
    out.theta0 += Utils::m_pi;
    out.kappa0 = -(out.kappa0);
    out.dk     = dk;
    while ( out.theta0 >  Utils::m_pi ) out.theta0 -= Utils::m_2pi;
    while ( out.theta0 < -Utils::m_pi ) out.theta0 += Utils::m_2pi;
  }

  // - - - - - - - - - - - - - - - - - - - - - - - - - - - - - - - - - - - - - -

  void
  ClothoidData::rotate( real_type angle, real_type cx, real_type cy ) {
    real_type dx  = x0 - cx;
    real_type dy  = y0 - cy;
    real_type C   = cos(angle);
    real_type S   = sin(angle);
    real_type ndx = C*dx - S*dy;
    real_type ndy = C*dy + S*dx;
    x0      = cx + ndx;
    y0      = cy + ndy;
    theta0 += angle;
  }

  void
  ClothoidData::origin_at( real_type s_origin ) {
    real_type C, S;
    real_type sdk = s_origin*dk;
    GeneralizedFresnelCS(
      sdk*s_origin,
      kappa0*s_origin,
      theta0,
      C, S
    );
    x0     += s_origin*C;
    y0     += s_origin*S;
    theta0 += s_origin*(kappa0+0.5*sdk);
    kappa0 += sdk;
  }

  // - - - - - - - - - - - - - - - - - - - - - - - - - - - - - - - - - - - - - -

  real_type
  ClothoidData::split_at_flex( ClothoidData & C0, ClothoidData & C1 ) const {
    // flex inside, split clothoid
    real_type sflex = -kappa0/dk;
    C0.theta0 = theta0 + 0.5*kappa0*sflex;
    eval( sflex, C0.x0, C0.y0 );
    C1.x0     = C0.x0;
    C1.y0     = C0.y0;
    C1.theta0 = C0.theta0+Utils::m_pi; // reverse curve
    C0.kappa0 = C1.kappa0 = 0;
    C0.dk     = C1.dk     = dk;
    return sflex;
  }

  // - - - - - - - - - - - - - - - - - - - - - - - - - - - - - - - - - - - - - -

  real_type
  ClothoidData::aplus( real_type dtheta ) const {
    real_type tmp = 2*dtheta*dk;
    real_type k0  = kappa0;
    if ( k0 < 0 ) { tmp = -tmp; k0 = -k0; }
    return 2*dtheta/(k0+sqrt(tmp+k0*k0));
  }

  // - - - - - - - - - - - - - - - - - - - - - - - - - - - - - - - - - - - - - -

  static real_type const one_degree = Utils::m_pi/180;

  bool
  ClothoidData::bbTriangle(
    real_type   L,
    real_type & xx0,
    real_type & yy0,
    real_type & xx1,
    real_type & yy1,
    real_type & xx2,
    real_type & yy2
  ) const {
    real_type theta_max = theta( L );
    real_type theta_min = theta0;
    real_type dtheta    = abs( theta_max-theta_min );
    if ( dtheta < Utils::m_pi_2 ) {
      real_type alpha, tx0, ty0;
      eval( 0, xx0, yy0 );
      eval_D( 0, tx0, ty0 );
      if ( dtheta > one_degree ) {
        real_type tx1, ty1;
        eval( L, xx1, yy1 );
        eval_D( L, tx1, ty1 );
        real_type det = tx1*ty0-tx0*ty1;
        alpha = ((yy1-yy0)*tx1 - (xx1-xx0)*ty1)/det;
      } else {
        // se angolo troppo piccolo uso approx piu rozza
        alpha = L;
      }
      xx2 = xx0 + alpha*tx0;
      yy2 = yy0 + alpha*ty0;
      return true;
    } else {
      return false;
    }
  }

  // - - - - - - - - - - - - - - - - - - - - - - - - - - - - - - - - - - - - - -

  bool
  ClothoidData::bbTriangle_ISO(
    real_type   L,
    real_type   offs,
    real_type & xx0,
    real_type & yy0,
    real_type & xx1,
    real_type & yy1,
    real_type & xx2,
    real_type & yy2
  ) const {
    real_type theta_max = theta( L );
    real_type theta_min = theta0;
    real_type dtheta    = abs( theta_max-theta_min );
    if ( dtheta < Utils::m_pi_2 ) {
      real_type alpha, tx0, ty0;
      eval_ISO( 0, offs, xx0, yy0 );
      eval_D( 0, tx0, ty0 ); // no offset solo scalato
      if ( dtheta > one_degree ) {
        real_type tx1, ty1;
        eval_ISO( L, offs, xx1, yy1 );
        eval_D( L, tx1, ty1 ); // no offset solo scalato
        real_type det = tx1*ty0-tx0*ty1;
        alpha = ((yy1-yy0)*tx1 - (xx1-xx0)*ty1)/det;
      } else {
        // se angolo troppo piccolo uso approx piu rozza
        alpha = L;
      }
      xx2 = xx0 + alpha*tx0;
      yy2 = yy0 + alpha*ty0;
      return true;
    } else {
      return false;
    }
  }

  // - - - - - - - - - - - - - - - - - - - - - - - - - - - - - - - - - - - - - -

  int
  ClothoidData::build_G1(
    real_type   _x0,
    real_type   _y0,
    real_type   _theta0,
    real_type   x1,
    real_type   y1,
    real_type   theta1,
    real_type   tol,
    real_type & L,
    bool        compute_deriv,
    real_type   L_D[2],
    real_type   k_D[2],
    real_type   dk_D[2]
  ) {
    static real_type const CF[] = {
      2.989696028701907,   0.716228953608281,
      -0.458969738821509, -0.502821153340377,
      0.261062141752652,  -0.045854475238709
    };

    x0     = _x0;
    y0     = _y0;
    theta0 = _theta0;

    // traslazione in (0,0)
    real_type dx   = x1 - x0;
    real_type dy   = y1 - y0;
    real_type r    = hypot( dx, dy );
    real_type phi  = atan2( dy, dx );
    real_type phi0 = theta0 - phi;
    real_type phi1 = theta1 - phi;

    phi0 -= Utils::m_2pi*round(phi0/Utils::m_2pi);
    phi1 -= Utils::m_2pi*round(phi1/Utils::m_2pi);

    if      ( phi0 >  Utils::m_pi ) phi0 -= Utils::m_2pi;
    else if ( phi0 < -Utils::m_pi ) phi0 += Utils::m_2pi;
    if      ( phi1 >  Utils::m_pi ) phi1 -= Utils::m_2pi;
    else if ( phi1 < -Utils::m_pi ) phi1 += Utils::m_2pi;

    real_type delta = phi1 - phi0;

    // punto iniziale
    real_type X  = phi0*Utils::m_1_pi;
    real_type Y  = phi1*Utils::m_1_pi;
    real_type xy = X*Y;
    Y *= Y; X *= X;
    real_type A = (phi0+phi1) * ( CF[0] + xy*(CF[1] + xy*CF[2]) +
                                  (CF[3]+xy*CF[4])*(X+Y) + CF[5]*(X*X+Y*Y) );
    // newton
    real_type g=0, dg, intC[3], intS[3];
    int_type  niter = 0;
    do {
      GeneralizedFresnelCS( 3, 2*A, delta-A, phi0, intC, intS );
      g   = intS[0];
      dg  = intC[2] - intC[1];
      A  -= g / dg;
    } while ( ++niter <= 10 && abs(g) > tol );

    UTILS_ASSERT(
      abs(g) <= tol,
      "Newton do not converge, g = {} niter = {}\n",
      g, niter
    );
    GeneralizedFresnelCS( 2*A, delta-A, phi0, intC[0], intS[0] );
    L = r/intC[0];

    UTILS_ASSERT( L > 0, "Negative length L = {}\n", L );
    this->kappa0 = (delta-A)/L;
    this->dk     = 2*A/L/L;

    if ( compute_deriv ) {

      real_type alpha = intC[0]*intC[1] + intS[0]*intS[1];
      real_type beta  = intC[0]*intC[2] + intS[0]*intS[2];
      real_type gamma = intC[0]*intC[0] + intS[0]*intS[0];
      real_type tx    = intC[1]-intC[2];
      real_type ty    = intS[1]-intS[2];
      real_type txy   = L*(intC[1]*intS[2]-intC[2]*intS[1]);
      real_type omega = L*(intS[0]*tx-intC[0]*ty) - txy;

      delta = intC[0]*tx + intS[0]*ty;

      L_D[0] = omega/delta;
      L_D[1] = txy/delta;

      delta *= L;
      k_D[0] = (beta-gamma-kappa0*omega)/delta;
      k_D[1] = -(beta+kappa0*txy)/delta;

      delta  *= L/2;
      dk_D[0] = (gamma-alpha-dk*omega*L)/delta;
      dk_D[1] = (alpha-dk*txy*L)/delta;
    }

    return niter;
  }

  #endif

  // - - - - - - - - - - - - - - - - - - - - - - - - - - - - - - - - - - - - - -

  #ifndef DOXYGEN_SHOULD_SKIP_THIS
  static
  real_type
  kappa_fun( real_type theta0, real_type theta ) {
    real_type x = theta0*theta0;
    real_type a = -3.714 + x * 0.178;
    real_type b = -1.913 - x * 0.0753;
    real_type c =  0.999 + x * 0.03475;
    real_type d =  0.191 - x * 0.00703;
    real_type e =  0.500 - x * -0.00172;
    real_type t = d*theta0+e*theta;
    return a * theta0 + b * theta + c * (t*t*t);
  }
  #endif

  // - - - - - - - - - - - - - - - - - - - - - - - - - - - - - - - - - - - - - -

  #ifndef DOXYGEN_SHOULD_SKIP_THIS
  static
  real_type
  theta_guess( real_type theta0, real_type k0, bool & ok ) {
    real_type x   = theta0*theta0;
    real_type a   = -3.714 + x * 0.178;
    real_type b   = -1.913 - x * 0.0753;
    real_type c   =  0.999 + x * 0.03475;
    real_type d   =  0.191 - x * 0.00703;
    real_type e   =  0.500 - x * -0.00172;
    real_type e2  = e*e;
    real_type dt  = d*theta0;
    real_type dt2 = dt*dt;
    real_type A   = c*e*e2;
    real_type B   = 3*(c*d*e2*theta0);
    real_type C   = 3*c*e*dt2 + b;
    real_type D   = c*(dt*dt2) + a*theta0 - k0;

    PolynomialRoots::Cubic cubicSolver( A, B, C, D );

    real_type r[3];
    int_type nr = cubicSolver.getRealRoots(r);

    // cerco radice reale piu vicina
    real_type theta;
    switch ( nr ) {
    case 0:
    default:
      ok = false;
      return 0;
    case 1:
      theta = r[0];
      break;
    case 2:
      if ( abs(r[0]-theta0) < abs(r[1]-theta0) ) theta = r[0];
      else                                       theta = r[1];
      break;
    case 3:
      theta = r[0];
      for ( int_type i = 1; i < 3; ++i ) {
        if ( abs(theta-theta0) > abs(r[i]-theta0) )
          theta = r[i];
      }
      break;
    }
    ok = abs(theta-theta0) < Utils::m_pi;
    return theta;
  }
  #endif

  // - - - - - - - - - - - - - - - - - - - - - - - - - - - - - - - - - - - - - -

  #ifndef DOXYGEN_SHOULD_SKIP_THIS
  bool
  ClothoidData::build_forward(
    real_type   x0,
    real_type   y0,
    real_type   theta0,
    real_type   kappa0,
    real_type   x1,
    real_type   y1,
    real_type   tol,
    real_type & L
  ) {
    // Compute guess angles
    real_type dx   = x1 - x0;
    real_type dy   = y1 - y0;
    real_type len  = hypot( dy, dx );
    real_type arot = atan2( dy, dx );
    real_type th0  = theta0 - arot;
    // normalize angle
    while ( th0 >  Utils::m_pi ) th0 -= Utils::m_2pi;
    while ( th0 < -Utils::m_pi ) th0 += Utils::m_2pi;

    // solve the problem from (0,0) to (1,0)
    real_type k0    = kappa0*len;
    real_type alpha = 2.6;
    real_type thmin = max(-Utils::m_pi,-theta0/2-alpha);
    real_type thmax = min( Utils::m_pi,-theta0/2+alpha);
    real_type Kmin  = kappa_fun( th0, thmax );
    real_type Kmax  = kappa_fun( th0, thmin );
    bool ok;
    real_type th = theta_guess( th0, max(min(k0,Kmax),Kmin), ok );
    if ( ok ) {
      for ( int_type iter = 0; iter < 20; ++iter ) {
        real_type LL, L_D[2], k_D[2], dk_D[2];
        build_G1(
          0, 0, th0,
          1, 0, th,
          tol, LL,
          true, L_D, k_D, dk_D
        );
        real_type f   = this->kappa0 - k0; // use kappa0 of the class
        real_type df  = k_D[1];
        real_type dth = f/df;
        th -= dth;
        if ( abs(dth) < tol && abs(f) < tol ) {
          // transform solution
          build_G1( x0, y0, theta0, x1, y1, arot + th, tol, L );
          return true;
        }
      }
    }
    return false;
  }

  // - - - - - - - - - - - - - - - - - - - - - - - - - - - - - - - - - - - - - -

  void
  ClothoidData::info( ostream_type & s ) const {
    fmt::print( s,
      "x0     = {}\n"
      "y0     = {}\n"
      "theta0 = {}\n"
      "kappa0 = {}\n"
      "dk     = {}\n",
      x0, y0, theta0, kappa0, dk
    );
  }

  // - - - - - - - - - - - - - - - - - - - - - - - - - - - - - - - - - - - - - -

  #endif
}

// EOF: Fresnel.cc
```

### Quick search

### Table of Contents

- Matlab Interface Manual
- C++ API
- MATLAB API

«
hide menu

menu
sidebar
»

### Navigation

- index
- toc
- Clothoids »
- Program Listing for File Fresnel.cc

© Copyright 2021, Enrico Bertolazzi and Marco Frego.
Created using Sphinx 4.2.0.
